# Expanded Regulatory T Cells Induce Alternatively Activated Monocytes With a Reduced Capacity to Expand T Helper-17 Cells

**DOI:** 10.3389/fimmu.2018.01625

**Published:** 2018-07-20

**Authors:** Marco Romano, Giorgia Fanelli, Nicole Tan, Estefania Nova-Lamperti, Reuben McGregor, Robert I. Lechler, Giovanna Lombardi, Cristiano Scottà

**Affiliations:** ^1^Immunoregulation Laboratory, MRC Centre for Transplantation, School of Immunology & Microbial Sciences, King’s College London, London, United Kingdom; ^2^Molecular and Translational Immunology Laboratory, Department of Clinical Biochemistry and Immunology, Faculty of Pharmacy, University of Concepcion, Concepcion, Chile

**Keywords:** regulatory T cells, immunoregulation, monocytes, alternatively activated macrophages, cell therapy

## Abstract

Regulatory T cells (Tregs) are essential in maintaining peripheral immunological tolerance by modulating several subsets of the immune system including monocytes. Under inflammatory conditions, monocytes migrate into the tissues, where they differentiate into dendritic cells or tissue-resident macrophages. As a result of their context-dependent plasticity, monocytes have been implicated in the development/progression of graft-vs-host disease (GvHD), autoimmune diseases and allograft rejection. In the last decade, Tregs have been exploited for their use in cell therapy with the aim to induce tolerance after solid organ transplantation and for the treatment of autoimmune diseases and GvHD. To date, safety and feasibility of Treg infusion has been demonstrated; however, many questions of how these cells induce tolerance have been raised and need to be answered. As monocytes constitute the major cellular component in inflamed tissues, we have developed an *in vitro* model to test how Tregs modulate their phenotype and function. We demonstrated that expanded Tregs can drive monocytes toward an alternatively activated state more efficiently than freshly isolated Tregs. The effect of expanded Tregs on monocytes led to a reduced production of pro-inflammatory cytokines (IL-6 and tumor necrosis factor-α) and NF-κB activation. Furthermore, monocytes co-cultured with expanded Tregs downregulated the expression of co-stimulatory and MHC-class II molecules with a concomitant upregulation of M2 macrophage specific markers, CD206, heme oxygenase-1, and increased interleukin-10 production. Importantly, monocytes co-cultured with expanded Tregs showed a reduced capacity to expand IL-17-producing T cells compared with monocyte cultured with freshly isolated Tregs and conventional T cells. The capacity to decrease the expansion of pro-inflammatory Th-17 was not cytokine mediated but the consequence of their lower expression of the co-stimulatory molecule CD86. Our data suggest that expanded Tregs have the capacity to induce phenotypical and functional changes in monocytes that might be crucial for tolerance induction in transplantation and the prevention/treatment of GvHD and autoimmune diseases.

## Introduction

Regulatory T cells (Tregs) maintain peripheral immunological tolerance by controlling autoreactive T cells and dampening inflammation (1). These cells represent 5–10% of all the circulating CD4^+^ T lymphocytes and constitutively express high level of CD25 and FOXP3 (2, 3). Tregs can be broadly divided in two main subpopulations: thymic derived Tregs (4) and peripherally induced Tregs generated by the stimulation of conventional T cells (Tconv) under specific tolerogenic conditions (5, 6). Tregs use a plethora of mechanisms to suppress the activation and proliferation of different immune cell subsets (7–9). The release of immunosuppressive cytokines such as interleukin-10 (IL-10) (10) and transforming grow factor-β (TGF-β) is essential for Treg function as they can modulate both T lymphocytes and antigen-presenting cells (APCs) activation (11). IL-10 can downregulate MHC-class II and co-stimulatory molecules on dendritic cells (DCs) (12, 13) and, at the same time, reduce the production of IL-6, IL-1β (14), and tumor necrosis factor-α (TNF-α) (15). However, in inflamed tissues, Tregs interact mainly with monocytes and macrophages. These cells are involved in both innate and adaptive immunity as they have the potential to phagocyte and kill bacteria, produce cytokines, and process/present antigen to lymphocytes (16–18). IL-10, together with TGF-β, can drive the differentiation of monocytes into M2 type c macrophages (19, 20). Compared with the classical M1 macrophages, these cells express high levels of the hemoglobin–haptoglobin scavenger receptor (CD163) and secrete less pro-inflammatory cytokines (21). Moreover, M2c can promote kidney repair *in vivo* by deactivating endogenous renal macrophages and by inhibiting CD4 T cells proliferation (20). Recently, it has been shown that IL-10 released by Tregs during the co-culture with monocytes, induced an upregulation of CD163 and CCL18 followed by reduced release of pro-inflammatory cytokines after LPS stimulation (22). In addition, IL-10 is involved in the control of genes implicated in the clearance of oxidative stress such as heme oxygenase-1 (HO-1) (23). This enzyme plays an essential role in suppressing immune responses during inflammation (24) autoimmune diseases (25) and allograft rejection (26).

Regulatory T cells can additionally exert their immunosuppressive function by contact-dependent mechanisms. They are the only T-cells that constitutively express cytotoxic T-lymphocyte antigen-4 (CTLA-4) (27). This molecule binds the same ligands as CD28, CD80, and CD86, thus limiting co-stimulatory signals during T cell activation. CTLA-4 can also downregulate DCs’ activity *via* trans-endocytosis of CD80 and CD86 resulting in diminished co-stimulation and T cell anergy (28).

In addition, the interaction between monocytes and Tregs *in vitro* induces the upregulation of the mannose scavenger receptor (CD206), a specific marker for M2a macrophages (22).

Current strategies for clinical management of transplant recipients and for the treatment of graft-vs-host disease (GvHD) involve the use of immunosuppressive drugs (29, 30). However, they do not fully prevent chronic graft rejection or GvHD and they are linked to morbidity and mortality. For this reason, Tregs have been extensively studied as therapeutic tool for the generation of tolerance in solid organ transplantation and for the treatment of autoimmune disorders and GvHD. Freshly isolated Tregs using Good Manufacturing Practice (GMP) protocols (31) have been infused in phase I clinical trials with no side effects (32–34). However, preclinical studies have also shown that expanded Tregs are more suitable in preventing graft rejection and GvHD than freshly isolated Tregs (35). We have recently developed a clinically applicable protocol for the expansion of human Tregs (36, 37) which involves the use of rapamycin and IL-2.

With the aim of better understanding the mechanisms adopted by expanded Tregs in the induction of tolerance, we have settled an *in vitro* model to study whether Tregs can induce an anti-inflammatory phenotype in monocytes. Monocytes display extreme plasticity in response to signals from the microenvironment and their presence in rejecting allograft tissue is associated with worse graft function and/or survival (38). We hypothesized that the modulation of monocytes by Tregs might be a key mechanism in the induction of tolerance. The data obtained here suggest that expanded human Tregs induce an alternative activation status in monocytes with the potential to support the long-term acceptance of an allograft or to reduce the high inflammatory status which is critical for the progression of GvHD and autoimmune diseases.

## Materials and Methods

### Cell Isolation and Expansion

Peripheral blood mononuclear cells (PBMCs) from healthy donors were obtained from anonymized human leukocyte cones supplied by the National Blood Transfusion Service (NHS blood and transplantation, Tooting, London, UK). Human studies were conducted in accordance with the Helsinki Declaration and approved by the Institutional Review Board of Guy’s Hospital (Reference 09/H0707/86). Informed consent was obtained from all healthy donors prior to enrollment into the study. PBMCs were isolated by lympholyte (1.077 g/cm^3^) gradient stratification (Lymphoprep; Axis-Shield, Norway). Subsequently, highly purified CD4^+^CD25^+^, CD4^+^CD25^−^, CD4 (from HLA-A2^+^ donors), and CD14^+^ cells (from HLA-A2^−^ donors) were isolated using specific immunomagnetic cell isolation Kits (Miltenyi Biotech, Germany) according to the manufacturer’s instructions. Freshly isolated T cells were frozen and used when needed. Tregs expansion has been executed as already published by us (36, 39). Briefly, cells were cultured in X-Vivo (Lonza, UK) supplemented with 5% of Human Serum AB Male (BioWest, France) and 100 nM of rapamycin (LC-Laboratories, USA). Cells were then activated with anti-CD3/CD28 beads (ratio bead:cell of 1:1; Invitrogen, UK). IL-2 (1,000 IU/mL; Proleukin, Novartis, UK) was added at day 4 post activation and replenished every 2 days. Cells were re-stimulated every 10–12 days and used after 36 days from the first activation (three rounds of stimulation). Expanded cells were frozen and used when needed.

### Flow Cytometry and Cytokine Evaluation

Freshly isolated Tregs, Tconv, and expanded Tregs have been phenotypically evaluated by flow cytometry using antibodies listed in Table S1 in Supplementary Material.

After detaching, monocytes were incubated with Human TruStain FcX™ (Fc Receptor Blocking Solution, BioLegend, USA) for 10 min and then stained with Live/Dead Yellow (Thermo Fisher Scientific, UK) and extracellular antibodies as listed in Table S1 in Supplementary Material for 30 min at 4°C. For intracellular staining, T cells were activated with PMA/ionomycin for 5 h at 37°C. Cells were then stained with, anti-HLA-A2 and CD45RO followed by cell permeabilization and intracellular staining using anti-IL-17 (BL168, BioLegend, USA), anti-IL-4 (8D4-8, Thermo Fisher Scientific, UK), and anti-IFN-γ (B27, Thermo Fisher Scientific, UK). Permeabilization was performed with the FOXP3/Transcription Factor Staining Buffer Set (Thermo Fisher Scientific, UK) 30 min/4°C according to the manufacturer’s instructions. Samples were acquired on LSR-Fortessa flow cytometer and files analyzed using Flow Jo 9.7.5 (Tree Star Inc., USA). Supernatants from activated T cells and monocytes were used to detect cytokines production using LEGENDplex Human Th-Cytokine Assay and Human panel 2 Cytokine Assay (BioLegend, USA) according to the manufacturer’s instructions. Cytokines were acquired on a FACSCanto II (BD Biosciences, USA). Data analysis was carried out on BioLegend’s LEGENDplex Data Analysis Software. TGF-β evaluation from monocytes and T cells has been performed using Human TGF-β1 Platinum ELISA kit (Thermo Fisher Scientific, UK).

### Suppression Assay

1 × 10^5^ CD4^+^CD25^−^ (T-effectors) were labeled with CFSE (2.5 µM; Molecular Probe, USA) and cultured alone or at different ratios with freshly isolated Tregs, Tconv, and expanded Tregs. Effector T cells were stimulated with anti-CD3/CD28 beads (Thermo Fisher Scientific, UK) in U-bottom 96-well plates and incubated at 37°C, 5% CO_2_ for 5 days. Data were acquired on LSR-Fortessa and analyzed with Flow Jo 9.7.5 software (Tree Star Inc., USA). Suppression of proliferation by Tregs was analyzed as previously described (40).

### Monocytes-T Cells Co-Culture

**Figure 1:**
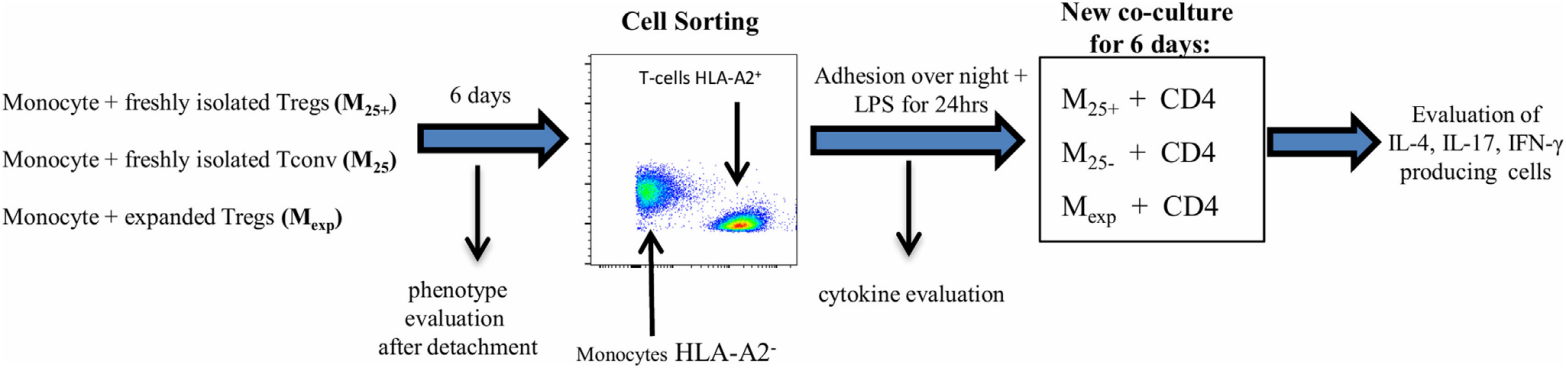
Experimental protocol. Co-culture experiments have been settled in 48-well plates pre-coated with anti-CD3 monoclonal antibody (50 ng/mL). 0.5 × 10^6^ monocytes (HLA-A2^−^) have been cultured in the presence of 0.25 × 10^6^ T cells (HLA-A2^+^) for 6 days. The resultant monocyte populations have been identified as M_25+_ [monocytes co-cultured with freshly isolated CD4^+^CD25^+^ regulatory T cells (Tregs)], M_25−_ [monocytes co-cultured with freshly isolated CD4^+^CD25^−^ conventional T cell (Tconv)], and M_exp_ (monocytes co-cultured with expanded Tregs). Cells were then detached and labeled with Live/Dead dying and HLA-A2 antibody for cell sorting following the gate strategy reported in Figure S1 in Supplementary Material. At the same time, the expression of CD86, CD14, CD80 CD40, CD206, CD163, and HLA-DR has been evaluated by flow cytometry. After sorting, 150 × 10^5^ cells have been lysed for WB analysis and 50 × 10^3^ cells per condition were placed in a new 48-well plate and left at 37°C overnight to ensure complete adhesion. Adhering cells have been stimulated with LPS for 24 h and then co-cultured, for other 6 days, with allogeneic CD4 T cells (10^5^ per condition, HLA-A2^+^ coming from the same donor of the Tconv/Treg) in presence of soluble anti-CD3 (50 ng/mL). Intracellular staining to evaluate IL-4, IL-17, and IFN-γ-producing CD4 T cells has been executed at the end of the co-culture. Supernatant after LPS stimulation (50 ng/mL) has been used for cytokine evaluation and for the experiment using the M_25−_ conditioned medium.

A co-culture experiment was settled in 48-well plates pre-coated with anti-CD3 monoclonal antibody (50 ng/mL; clone UCHT1; BioLegend, USA). 0.5 × 10^6^ monocytes (HLA-A2^−^) have been co-cultured in presence of 0.25 × 10^6^ T cells (HLA-A2^+^) for 6 days as detailed in Figure 1. The resultant monocyte populations have been identified as M_25+_ (monocytes co-cultured with freshly isolated Tregs), M_25−_ (monocytes co-cultured with Tconv), and M_exp_ (monocytes co-cultured with expanded Tregs). Cells were then detached using Accutase (STEMCELL Technologies, UK) and labeled with Live and Dead dying (Thermo Fisher Scientific, UK) and HLA-A2 antibody (Miltenyi, Germany) for sorting, following the gate strategy reported in Figure S1 in Supplementary Material. After sorting, 50 × 10^3^ cells per condition were placed in a new 96-well plate and left at 37° overnight to ensure a complete adhesion. Adhering cells have been stimulated 24 h with LPS (50 ng/mL) and then co-cultured, for other 6 days, with allogeneic CD4 T cells (10^5^ per condition, HLA-A2^+^) in the presence of soluble anti-CD3 (clone OKT3 50 ng/mL; eBioscience). To understand the mechanisms behind the monocytes/CD4 interaction, M_25−_ have been cultured for 6 days in presence/absence of tocilizumab (100 ng/mL, Roche UK) infliximab (100 ng/mL, Napp Pharmaceutical group, UK) and abatacept (Orencia^®^ Bristol Mayer Squibb, USA). Specifically, to ensure a complete block of TNF-α and the co-stimulation during the co-culture, M_25−_ have been pre-incubated with Abatacept and Infliximab for at least 1 h; similar to selectively block the IL-6 receptor only on T cells, CD4 have been pre-incubated with Tocilizumab. Drugs have been replenished after 3 days.

To evaluate the effects of cytokines released by monocytes on T cell activation, 1 × 10^5^ CD4^+^ T cells have been activated using anti-CD3/CD28 microbeads (beads to cells ratio 1:4) (Thermo Fisher Scientific, UK) in X-vivo and M_25−_ conditioned medium.

### Western Blot Analysis

Sorted cells were pelleted and lysed with cold RIPA buffer (Thermo Fisher Scientific, UK) containing protease inhibitors 1× (Calbiochem, Germany). Protein lysates were denaturated by adding 2× laemmli buffer (Bio-Rad, USA) containing 5% β-mercaptoethanol (Sigma-Aldrich, Germany). Protein samples were separated on 10% SDS polyacrylamide gels and transferred onto polyvinylidene difluoride membranes (Millipore, USA). Membranes were blocked in 5% non-fat dry milk (Bio-Rad, USA) in PBS 0.1% Tween-20 for 1 h at room temperature and incubated with phospho- and total-p65 (Ser536) (both from Cell Signaling Technology, USA), HO-1 (Abcam, UK), and β-actin (Santa Cruz Biotechnology, USA) antibodies overnight at 4°C. Proteins were detected with chemiluminescence detection reagents (Bio-Rad, USA) after HRP conjugated secondary antibody (Cell Signaling Technology, USA) incubation using ImageQuant LASS4000 mini (GE Healthcare Life Science, UK) and quantified using Image Studio Lite version 5.2 (LI-COR Biosciences, USA).

### Statistical Analysis

Statistical analyses were performed using Prism Version 7 software (Graph-Pad, USA). Data were expressed as mean ± SEM where applicable using bar charts. Unpaired *t*-test has been used to evaluate different T cell subsets. One-way ANOVA followed by Tukey test was used for monocytes experiment and all *p* values were considered significant when ≤0.0.05. Specifically for *p* values: *p < 0.05, **p < 0.01, and ***p < 0.001.

## Results

### Expansion of Tregs Using Rapamycin Increases Their Tolerogenic Functions

To test the effects of expanded Tregs on monocytes, CD4^+^CD25^+^ were isolated and expanded as detailed in Section “Materials and Methods.” At the end of the culture, we evaluated the phenotype and function of expanded Tregs compared with freshly isolated Tconv and Tregs.

**Figure 2:**
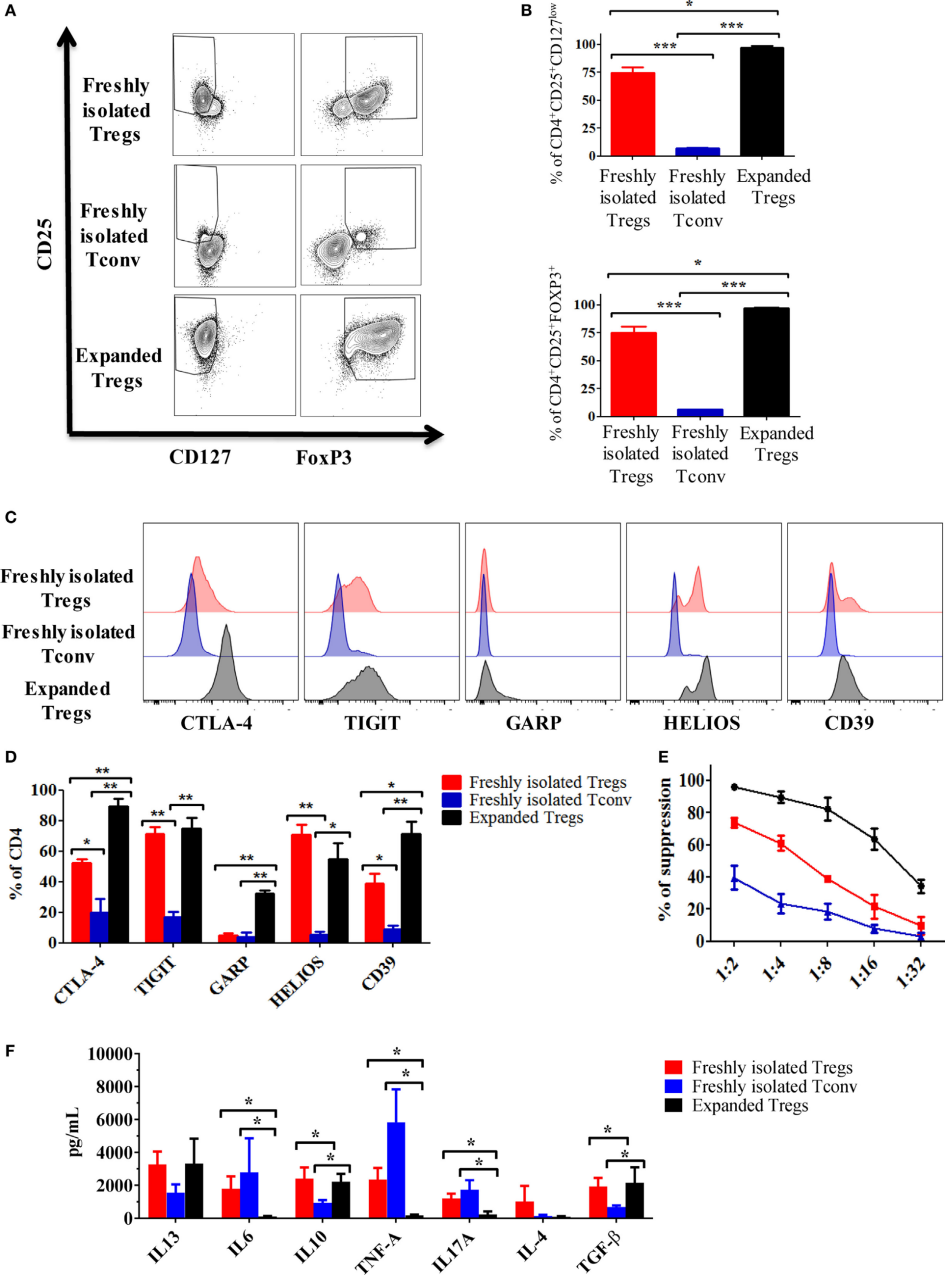
Expansion of regulatory T cells (Tregs) using rapamycin increases their immunosuppressive capacity. **(A)** Representative dot plots showing the expression of CD25 vs FOXP3 and CD127 in expanded Tregs, freshly isolated Tregs and conventional T cell (Tconv). **(B)** Cumulative data of six independent experiments showing the percentages of CD4^+^CD25^+^CD127^low^ and CD4^+^CD25^+^FOXP3^+^ in expanded Tregs, freshly isolated Tregs and Tconv. Representative histogram **(C)** and cumulative data **(D)** of six independent experiments showing the expression of cytotoxic T-lymphocyte antigen-4 (CTLA-4), TIGIT, GARP, HELIOS, and CD39 in expanded Tregs, freshly isolated Tregs and Tconv. Data are expressed as percentage of expression of CD4^+^ cells. **(E)** Suppressive ability at different ratios of expanded Tregs, freshly isolated Tregs and Tconv vs third party Teff. Means of six independent experiments are expressed as percentage of inhibition of the Teff proliferation. **(F)** Cytokine production by expanded Tregs, freshly isolated Tregs and Tconv after 3 days stimulation with anti-CD3/CD28 beads (4:1 cells to beads ratio). Data are expressed as mean of six independent experiments. In all the experiments, data are presented as mean ± SEM and analyzed using unpaired *t-*test with *p < 0.05, **p < 0.01, and ***p < 0.001.

As shown in Figures 2A,B, the percentage of CD4^+^CD25^+^CD127^low^ and CD4^+^CD25^+^FOXP3^+^ was increased after expansion (74.7 ± 4.9 vs 97.2 ± 1.2 and 75.3 ± 5.1 vs 97.3 ± 0.5, respectively). Noteworthy, in this study, we used a protocol mirroring the GMP Treg isolation showing the presence of a small fraction of cells negative for FOXP3 and positive for CD127. Similarly, we were able to detect FOXP3-positive cells in the Tconv fraction (Figures 2A,B).

The phenotypic analysis of freshly isolated Tregs, Tconv, and expanded Tregs showed an increased expression of functional markers such as CTLA-4, CD39, and GARP especially on expanded Tregs (Figures 2C,D). No statistically significant differences were found in the expression of HELIOS and TIGIT between freshly isolated and expanded Tregs. However, in comparison with Tconv, both freshly isolated and expanded Tregs expressed high level of these two markers (Figures 2C,D).

To further characterize the Tregs, we tested their suppressive ability. As expected, expanded Tregs showed the highest capacity to inhibit the proliferation of co-cultured CFSE-labeled effector cells when compared with freshly isolated Tregs and Tconv (Figure 2E).

Finally, to establish the cytokine profile produced by the different cell preparations, we activated freshly isolated Tregs, expanded Tregs, and Tconv *in vitro* for 3 days using anti-CD3/CD28 beads. Compared with freshly isolated Tregs and Tconv, expanded Tregs produced less IL-6, IL-17, and TNF-α (Figure 2F). Furthermore, both freshly isolated and expanded Tregs produced more IL-10 and TGF-β compared with Tconv (Figure 2F). Although no statistically significant differences have been found, both freshly isolated and expanded Tregs tended to release more IL-13 than Tconv.

Overall, these results confirmed that expanded Tregs have an increased capacity to generate tolerance in comparison to freshly isolated Tregs and Tconv.

### Expanded Tregs Differentially Activate Monocytes Inducing a Unique Population

**Figure 3:**
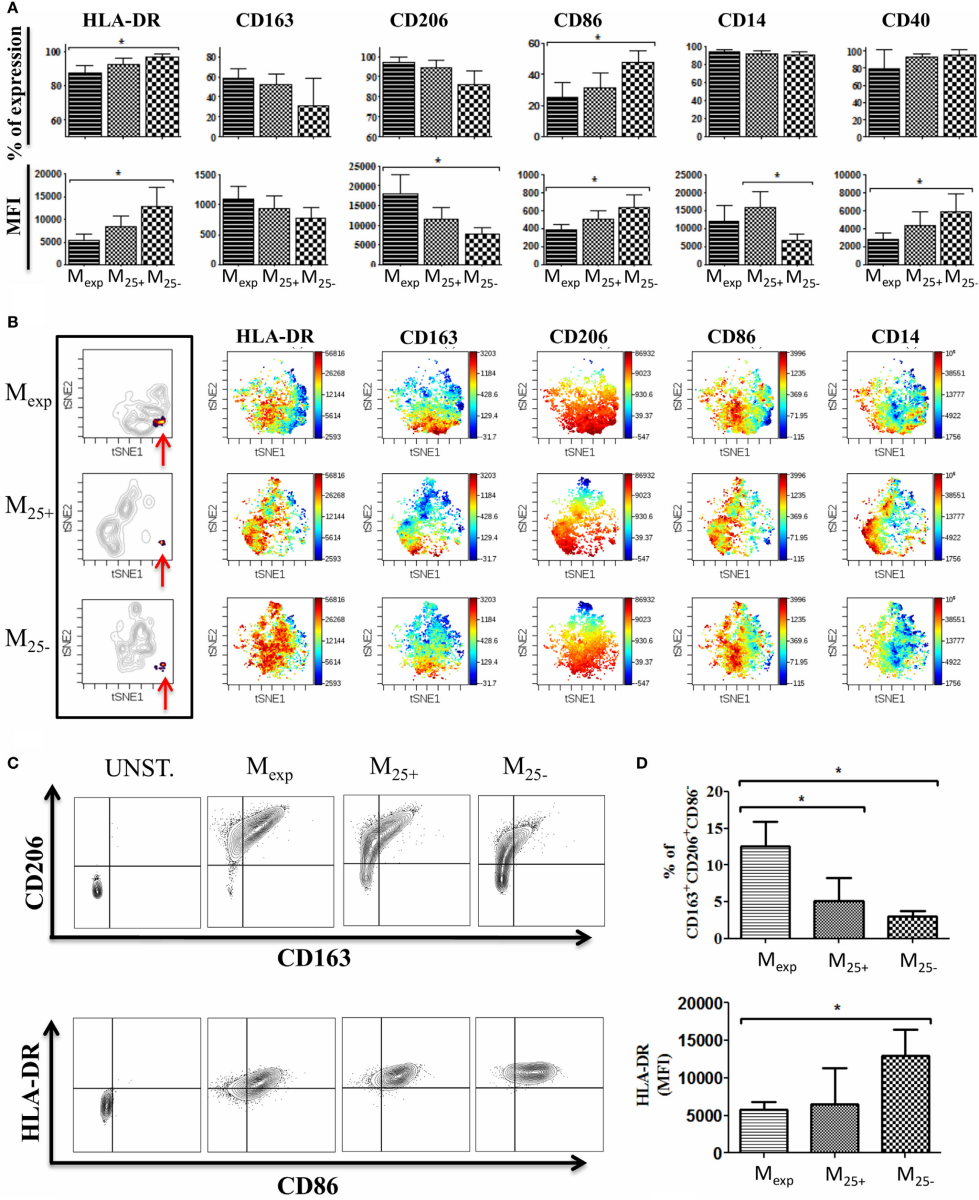
Phenotype of monocytes after co-culture with T cells. **(A)** Expression of HLA-DR, CD86, CD14, CD206, CD163, and CD40 evaluated by flow cytometry on monocytes co-culture with expanded regulatory T cells (Tregs) (M_exp_), freshly isolated Tregs (M_25+_), and freshly isolated conventional T cell (M_25−_) for 6 days. Percentages of expression (upper panels) and MFI (lower panels) of 10 independent experiments. **(B)** Visualization of one representative automated clustering method including t-distributed stochastic neighbor embedding (t-SNE) highlighting the unique cluster (indicated by the red arrows) in M_exp_, M_25+_, and M_25−_ followed by the intensity of expression of HLA-DR, CD86, CD14, CD206, and CD163. **(C)** Representative plots showing CD206 and CD163 expression (upper panels) and HLA-DR plus CD86 on CD206^+^DC163^+^ cells (lower panel) in unstained control, M_exp_, M_25+_, and M_25−_ cells. **(D)** Cumulative data of six independent experiments showing the percentages of CD14^+^CD206^+^CD163^+^CD86^−^ in M_exp_, M_25+_, and M_25−_ (upper panel) and their HLA-DR expression (lower panel). In all the experiments, data, presented as mean ± SEM, were analyzed using one-way ANOVA followed by Tukey with *p < 0.05.

To test the capacity of Tregs to activate/modulate CD14^+^ monocytes, we co-cultured HLA-A2^−^ monocytes with HLA-A2^+^ expanded Tregs, freshly isolated Tregs, and Tconv as detailed in Figure 1. We evaluated the impact of this interaction by measuring on monocytes the expression of CD80, CD86, CD14, CD206, CD163, CD40, and HLA-DR at day 0 and after 6 days of culture (Figure S2 in Supplementary Material; Figure 3A). Monocytes co-cultured with expanded Tregs (M_exp_) expressed less HLA-DR both as percentage and MFI compared with monocytes co-cultured with Tconv (M_25−_). Freshly isolated Tregs showed the capacity to reduce HLA-DR expression on monocytes (M_25+_), but not to the same extent than expanded Tregs. Of note, no significant differences were found in the expression of HLA-DR between freshly isolated and cultured monocytes (Figure S2 in Supplementary Material; Figure 3A).

As Tregs have the potential to produce cytokines involved in the differentiation of M2 macrophages, we measured the expression of M2 specific markers such as CD206 and CD163. After 6 days of co-culture, the expression of these markers was increased in all the conditions when compared with the freshly isolated cells. However, monocytes co-cultured with either freshly isolated Tregs or expanded Tregs tended to upregulate the expression of CD163 more than M_25−_, while M_exp_ showed the highest levels (MFI) of CD206.

The analysis of the co-stimulatory molecules revealed a reduction of CD86 expression in M_25+_, M_25−_, and M_exp_ compared with freshly isolated monocytes. In detail, the comparison between M_25+_, M_25−_, and M_exp_ showed that the highest expression of CD86 was on M_25−_ (Figure 3A). Conversely, CD40 was increased in all the conditions when compared with freshly isolated monocytes and M_25−_ showed the highest MFI (Figure 3A).

CD80 expression was not detected in all conditions before and after co-culture (data not shown). Of note, all monocytes from the co-cultures were positive for CD14, although in M_25−_ CD14 MFI was significantly reduced compared with M_25+_ (Figure 3A).

We then performed an automated clustering method including t-distributed stochastic neighbor embedding to use SNE visually (viSNE) as a mean of identifying different cell populations (41). Using the most representative markers commonly used to discriminate M2 macrophages (CD86, CD14, CD206, CD163, and HLA-DR), we showed that M_25+_, M_25−_, and M_exp_ clustered individually, confirming that expanded Tregs differentially activate monocytes compared with Tconv and freshly isolated Tregs (Figure 3B). In particular, we were able to detect a unique cluster (Figure 3B) which included CD14-positive cells expressing high levels of CD206, CD163 with low or no expression of CD86 and HLA-DR (Figure 3B). These data were confirmed by flow cytometry (Figures 3C,D) as the percentage of CD14^+^CD206^+^CD163^+^CD86^−^ cells was 5.15 ± 1.26, 3.07 ± 0.7, and 14 ± 3.4 in M_25+_, M_25−_, and M_exp_ respectively. In line with viSNE analysis, the MFI for HLA-DR on CD14^+^CD206^+^CD163^+^CD86^−^ was significantly reduced when monocytes were co-cultured with expanded Tregs (Figure 3D). Similar to what we have shown by analyzing the monocyte populations as a whole, expanded Tregs were more powerful in reducing HLA-DR than freshly isolated Tregs.

In conclusion, we showed that the interaction of expanded Tregs with monocytes is more powerful in reducing co-stimulatory and MHC-class II molecules and in the upregulation of M2 macrophages specific markers.

### M_exp_ Showed a Reduced Activation Status Compared With M_25+_ and M_25−_

**Figure 4:**
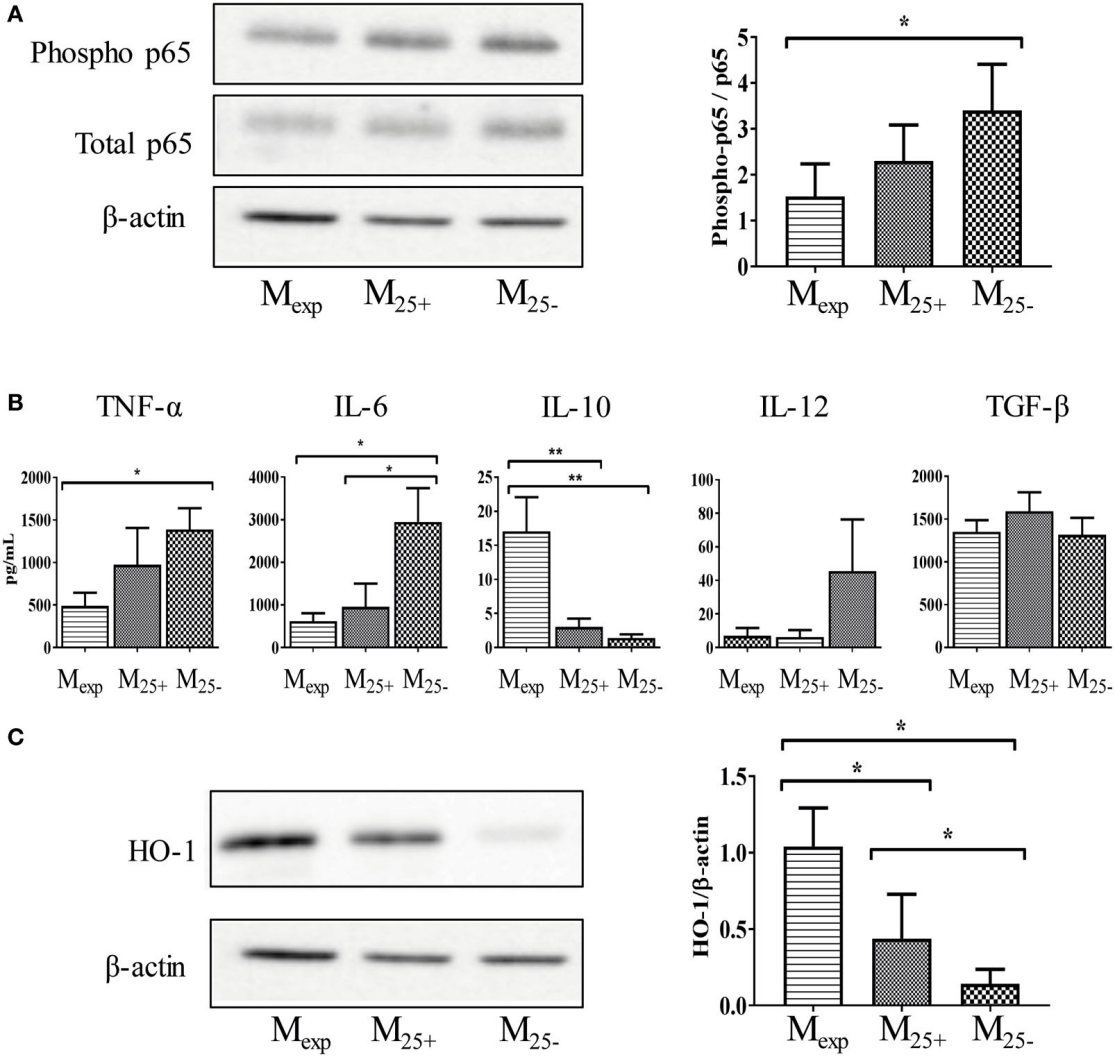
M_exp_ showed a reduced activation status compared with M_25+_ and M_25−_. **(A)** Representative WB showing the expression of phosphorylated and total p65 and β-actin in M_exp_, M_25+_, and M_25−_ (left panels); cumulative data of four independent experiments showing the ratio between phosphorylated and total p65 (right panel) in M_exp_, M_25+_, and M_25−_ cells. **(B)** Cytokine production of sorted M_exp_, M_25+_, and M_25−_ after LPS stimulation (50 ng/mL) for 24 h. **(C)** Representative WB showing the expression of β-actin and heme oxygenase-1 (HO-1) in M_exp_, M_25+_, and M_25−_ (left panels); cumulative data of four independent experiments showing the ratio between HO-1 and β-actin (right panel) in M_exp_, M_25+_, and M_25−_ cells. In all the experiments, data, presented as mean ± SEM, were analyzed using one-way ANOVA followed by Tukey with *p < 0.05, **p < 0.01. Full length-blots are presented in Figure S4 in Supplementary Material. The same representative β-actin blot is shown in panels **(A,C)**.

After activation, monocytes orchestrate both the initiation and resolution of inflammation mediating either pro-inflammatory or anti-inflammatory immune responses. For this reason, we investigated the activation status of M_25+_, M_25−_, and M_exp_ cells. In monocytes, NF-κB is an important transcriptional factor linked to the production of pro-inflammatory cytokines and cell surface receptors (42). Therefore, we evaluated the phosphorylation of NF-κB in sorted cells at the end of the co-culture as described in Figure 1. As shown in Figure 4A, we found a decreased activation of NF-κB in monocytes co-cultured with Tregs, and this effect was more pronounced in those cells cultured with expanded Tregs. Indeed, NF-κB (p65) phosphorylation was significantly reduced in M_exp_ compared with M_25−_, although no statistical difference was observed between M_exp_ and M_25+_ (Figure 4A).

Due to the different activation status of monocytes, we then evaluated their capacity to produce cytokines in response to LPS stimulation. As shown in Figure 4B, M_exp_ had a reduced capacity to secrete TNF-α compared with M_25−_, while IL-6 and IL-12 were reduced in both M_25+_ and M_exp_ compared with M_25−_. Conversely, M_exp_ produced more IL-10 in comparison to either M_25−_ or M_25+_. Other cytokines such as IL-1α, IL-1β, IL-18, and TLSP were not detected in any condition (data not shown). Similarly, no differences were found in the production of TGF-β (Figure 4B).

As IL-10 controls genes implicated in the clearance of oxidative stress, we evaluated the expression of HO-1. This enzyme catalyzes heme degradation into carbon monoxide (CO), ferrous iron, and biliverdin. These cyto-protective molecules have antioxidant and anti-inflammatory properties, and they have been linked to both GvHD prevention (43) and induction of tolerance (26). As expected, HO-1 expression was upregulated in M_exp_ compared with M_25+_ and M_25−_ (Figure 4C).

Here, we showed that expanded Tregs reduced monocytes activation and the release of pro-inflammatory cytokines. In addition, they increase the expression of molecules (IL-10 and HO-1) able to inhibit inflammation.

### M_exp_ Reduced the Generation of IL-17-Producing Cells due to Their Lower CD86 Expression

**Figure 5:**
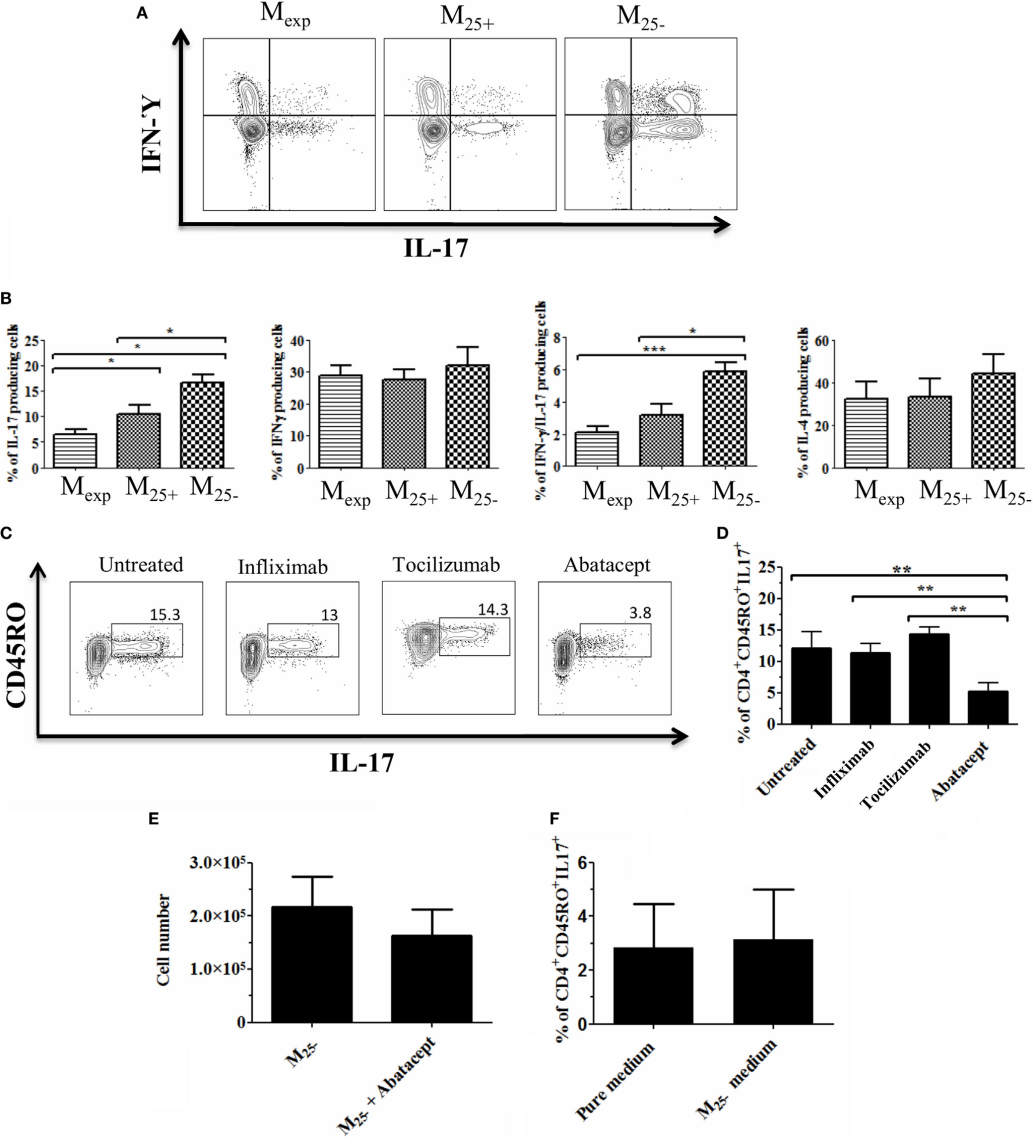
M_exp_ reduced the generation of IL-17-producing cells due to their lower CD86 expression. **(A)** Representative plots showing the percentages of IL-17, IL-17/IFN-γ, and IFN-γ-producing CD4^+^ cells after co-culture with M_exp_, M_25+_, and M_25−_. **(B)** Cumulative data of nine independent experiments showing the percentages of IL-17, IL-17/IFN-γ, and IFN-γ and IL-4 producing CD4^+^ cells. Representative plots **(C)** and cumulative data **(D)** of four independent experiments showing the percentages of memory IL-17-producing CD4^+^ cells after co-culture with M_25−_ alone or in the presence of infliximab, tociluzmab, and abatacept. **(E)** Cell count of CD4^+^ cells (*n* = 4) co-cultured with M_25−_ alone or in the presence of Abatacept. **(F)** Cumulative data of four independent experiments showing the percentages of memory IL-17-producing CD4^+^ stimulated with anti-CD3/28 beads in medium alone or M_25−_ conditioned medium. In all the experiments, data, presented as mean ± SEM, were analyzed using one-way ANOVA followed by Tukey with *p < 0.05, **p < 0.01.

Although DCs are considered the main APCs able to activate and drive the differentiation of T helper cells, monocytes/macrophages can play a similar role (44). To test the capacity of M_25+_, M_25−_, and M_exp_ monocytes to influence T helper differentiation/proliferation, sorted cells (Figure S3 in Supplementary Material) were co-cultured with CD4^+^ T cells as described in Figure 1. After 6 days of co-culture, the percentages of IL-4, IL-17, IFN-γ, and IL-17/IFN-γ-producing cells were evaluated. As shown in Figures 5A,B, both freshly isolated and expanded Tregs reduced the percentage of IL-17 and IL-17/IFN-γ-producing cells compared with Tconv. Importantly, M_exp_ were more powerful in reducing the percentages of IL-17-producing cells compared with M_25+_ (10.9 ± 1.7 vs 6.6 ± 0.8%, respectively). No statistically significant differences were found in the percentages of IL-4 and IFN-γ-producing cells. Finally, in none of the conditions, we could detect a stable induction of Tregs (data not shown).

The differentiation of IL-17-producing cells is mediated by IL-23, IL-1β, TNF-α, and IL-6 (45); however, this is not solely cytokine dependent (46). As M_exp_ produced low levels of IL-6 and TNF-α with a reduced expression of co-stimulatory molecules, we co-cultured M_25−_ and CD4^+^ T cells together with factors inhibiting either cytokines or co-stimulatory molecules expression. Three different blocking monoclonal antibodies were used in this experiment: infliximab (anti-TNF-α), tocilizumab (anti-IL-6 receptor), and abatacept (CTLA-4 fusion protein used to block CD80/CD86 co-stimulation). We found that the capacity of M_25−_ to increase IL-17-producing cells was drastically reduced only in the presence of abatacept (Figures 5C,D). Of note, this was not due to cell death as the numbers at the end of the culture were comparable between CD4^+^ T cells co-cultured with M_25−_ alone or in the presence of abatacept (Figure 5E). To further confirm the role of co-stimulation in the expansion of IL-17-producing T cells in our experimental condition, CD4^+^ T cells were stimulated with anti-CD3/CD28 beads in medium coming from M_25−_ culture (conditioned medium). No difference was observed between the two culture conditions confirming the need for cell:cell contact in Th-17 proliferation (Figure 5F).

Overall, our data suggest that M_exp_ had a reduced capacity to generate/expand IL-17- and IL-17/IFN-γ-producing T cells due mainly to their lower CD86 expression.

## Discussion

Over the past years, Tregs moved from promising cell candidates for tolerance induction to a therapeutic tool for the treatment of GvHD (47), autoimmune disorders (48), or to induce transplantation tolerance (49). Results from the first clinical trials showed that Tregs could be purified (47), expanded in GMP facilities (50), and re-infused in patients (32, 51). To date, the GMP immunomagnetic Treg isolation (CliniMACS) does not allow the purification of a highly pure FOXP3^+^ Treg product (47) as the cell fraction is contaminated with activated Tconv. To avoid the infusion of activated cells, we and others have developed Treg expansion protocols that include rapamycin (36, 52). These protocols have been demonstrated to be a successful approach for large-scale generation of functionally potent and phenotypically stable Tregs. Up to this stage, our group has positively completed two clinical trials investigating the safety of infusing *ex vivo* expanded Tregs in solid organ transplantation (the ONE Study and Thrill in kidney and liver transplantation, respectively). The outcomes of these trials are very promising; however, many questions remain unanswered, including which cells are targeted by Tregs *in vivo*.

Our expansion protocol allowed the proliferation of FOXP3^+^ cells reducing contaminants from the freshly isolated fraction. Consistently, here we have shown that expanded Tregs do not produce TNF-α, IL-6, and IL-17 and, at the same time, release high level of IL-10 and TGF-β. Expanded Tregs also express high level of functional markers like CTLA-4, GARP, and CD39. Furthermore, rapamycin expanded Tregs are more powerful than freshly isolated Tregs in driving monocytes differentiation toward alternatively activated macrophages (AAMs). In 2007, Tiemessen et al. described the ability of freshly isolated Tregs to drive monocytes toward AAMs (22). The authors showed that the co-culture of Tregs with monocytes upregulated M2-specific markers, reduced their NF-κB activation, and the release of pro-inflammatory cytokines. In line with these results, we found that expanded Tregs are more efficient than freshly isolated cells in doing so.

Tumor necrosis factor-α is crucial in all the phases of GvHD pathophysiology (53), and high concentrations of circulating TNF-α are considered as an immunological marker of graft rejection (54). CD14^+^ monocytes from healthy control cultured in the presence of TNF-α and GM-CSF differentiates into mature DCs or activated macrophages. Mature DCs expressed higher level of CD86 compared with those generated by GM-CSF and IL-4. As a consequence, these cells induced resting CD4 T cells to secrete IL-17. This evidence suggests that the priming of monocytes with TNF-α influences Th-17 responses induced by monocyte-derived mature DC (55). In our study, expanded Tregs decreased the expression of CD86 on monocytes during the co-culture. This effect might depend on the lack of TNF-α production, concomitant with the high level of IL-10 released by expanded Tregs. In addition, the presence of high level of CTLA-4 on these cells might also play a role in mediating the active removal of CD86 from cell surface. Importantly, the low levels of CD86 found in M_exp_ are essential in reducing their capacity to induce IL-17-producing T cells, as demonstrated in the co-culture of M_25−_ and CD4^+^ T cells where IL-6, TNF-α, and the co-stimulatory molecules were blocked. Th-17 have a detrimental effect in solid organ transplantation and GvHD (56, 57). Recently, we published that spontaneously kidney-tolerant recipients exhibited reduced Th-17 responses compared with patients with chronic rejection and healthy individuals. These data suggested that defective pro-inflammatory Th-17 responses might contribute to the maintenance of a stable graft function in the absence of immunosuppressive agents (58). In addition, a recent study showed that monocytes isolated from patients with acute or chronic GvHD induced more Th-17 cells *in vitro* compared with monocytes from patients without GvHD and healthy donors (33). Altogether, these data confirmed that controlling IL-17-producing T cells might be crucial for the induction of tolerance in transplantation and for the treatment of GvHD. Finally, TNF-α is also responsible for the activation of NF-κB (59). Therefore, the reduced production of TNF-α by expanded Tregs may explain the decreased phosphorylation of NF-κB that, in turn, lead to a reduced capacity to produce pro-inflammatory cytokines in M_exp_ compared with M_25−_.

In 2007, Tiemessen et al. hypothesized that the upregulation of CD206 by Tregs is contact dependent (22). In fact, when monocytes and Tregs were cultured together in the same well, but kept separated by a permeable support, monocytes were not able to upregulate CD206. However, in 2014, Fernando et al. (60) showed that during the *in vitro* differentiation of monocytes in M2a macrophages using IL-4 and IL-13, IL-6 enhanced the expression of CD206 in monocytes. In our culture conditions, expanded Tregs did not produce IL-6 compared with freshly isolated Tregs and Tconv reinforcing the idea that CD206 induction Treg-mediated might be controlled by a contact dependent mechanism. However, further investigations are needed to fully understand how T cells control the expression of CD206.

Both freshly isolated and expanded Tregs produced high level of IL-10 and TGF-β compared with Tconv; these anti-inflammatory cytokines have been linked with monocyte conversion to M2 type c macrophages (19). Our results showed consistently that both M_25+_ and M_exp_ had a reduced expression of CD86, CD40, and HLA-DR compared with M_25−_, although statistically significant results were only obtained in the co-culture with expanded Tregs. This is probably due to the production of pro-inflammatory cytokines, such as TNF-α (released by the freshly isolated Treg fraction), which has been shown to induce CD40 and CD86 in monocytes/macrophages. Another protein upregulated in M_exp_ compared with M_25+_ and M_25−_ was HO-1. This enzyme has an essential role in suppressing immune responses linked to inflammation (24), autoimmune diseases (25), and allograft rejection (26). Specifically, the pre-treatment of pancreatic allografts prior to transplantation with an HO-1 inducer increased graft survival due to the reduction of pro-inflammatory and an increase in anti-inflammatory cytokines (61). HO-1 has a role in GvHD prevention as well; the high HO-1 expression in GvHD target organs may attenuate the acute phase of the disease through the regulation of the balance between Th-17 and Tregs (43). The role of HO-1 in macrophage polarization has been demonstrated (62), with M2 macrophages expressing high levels of this enzyme. HO-1 expression can be regulated by IL-10 signaling and *vice versa*. Specifically, IL-10-mediated induction of HO-1 has been shown to require activation of STAT-3 and PI3K pathways in macrophage cell lines (62); subsequently, CO, the HO-1 bio-product, increases IL-10 production. In our model, monocytes treated with Tregs, upregulated HO-1 expression. In particular, M_exp_ were the cells with the highest HO-1 expression and the highest IL-10 production. This is in line with the positive feedback circuit between HO-1 and IL-10 reported in literature. These findings together with the reduced level of NF-κB phosphorylation might explain the observed reduced capacity of M_exp_ to produce IL-6 and TNF-α.

Overall, we have shown the capacity of expanded Tregs to alternatively activate monocytes/macrophages by contact and no-contact dependent mechanisms. Importantly and relevant to the *in vivo* impact of Treg therapy, expanded Tregs induced a subset of monocytes/macrophages that are characterized by a unique signature. This might explain why we were able to detect the CD14^+^CD206^+^CD163^+^CD86^−^ cell population with low HLA-DR expression in the M_exp_ group.

In conclusion, the capacity of expanded Tregs to induce AAMs might shed light on the mechanisms adopted by expanded Tregs to favor tolerance *in vivo*. Our cell therapy protocol aims to modulate the ratio between activated effector cells and Tregs. Increasing the level of Tregs over the effector T cells might drive the monocytes differentiation toward a population producing low level of TNF-α and IL-6 and high level of IL-10. Furthermore, the Treg-induced monocytes might be able to clear, by HO-1, the inflammation-mediated oxidative stress and contribute to reduce the infiltrating IL-17-producing cells responsible of graft rejection and GvHD progression. In conclusion, our *in vitro* model showed how expanded Tregs may favor the organ acceptance or reduce chronic inflammation. This might be exerted directly, inducing AAMs and/or indirectly through the reduction of IL-17-producing cells.

## Ethics Statement

Peripheral blood mononuclear cells were obtained from anonymized human leukocyte cones supplied by the National Blood Transfusion Service (NHS blood and transplantation, Tooting, London, UK). Human studies were conducted in accordance with the Helsinki Declaration and approved by the Institutional Review Board of Guy’s Hospital (Reference 09/H0707/86). Informed consent was obtained from all healthy donors prior to enrolment into the study.

## Author Contributions

MR conception design, collection and assembly data, data analysis and interpretation, and manuscript writing. GF, NT, RM, and EN-L collection and assembly data, data analysis, and interpretation. RL intellectual input and critical revision of the article. GL and CS conception and design, critical revision of the article for important intellectual contents.

## Conflict of Interest Statement

The authors declare that the research was conducted in the absence of any commercial or financial relationships that could be construed as a potential conflict of interest.
