# Supplementary material for: Expanded Regulatory T Cells Induce Alternatively Activated Monocytes With a Reduced Capacity to Expand T Helper-17 Cells
Source: Front Immunol. 2018 Jul 20;9:1625. doi: 10.3389/fimmu.2018.01625 (PMC6062605; doi:10.3389/fimmu.2018.01625)

Supplementary Material

Expanded regulatory T cells induce alternatively activated monocytes with a reduced capacity to expand T helper-17 cells

Marco Romano[1], Giorgia Fanelli[1], Nicole Tan[1], Estefania Nova-Lamperti[1,2], Reuben McGregor[1], Robert I. Lechler[1], Giovanna Lombardi[1]* and Cristiano Scottà[1]*

Correspondence: Cristiano Scottà M.Sc., Ph.D.

Tel: 020 7188 9738

Fax: 020 7188 7675

Email: cristiano.scotta@kcl.ac.uk

1 Supplementary Table 1

List of the antibodies used to characterize monocytes and T cells

**Antigen**	**Clone**	**Product Company**
HLA-A^2	REA517	Miltenyi Biotec, Germany
CD4	OKT4	Thermo Fisher Scientific, UK
CD25	4E3	Thermo Fisher Scientific, UK
CD127	A019D5	BioLegend, USA
FOXP3	PCH101	Thermo Fisher Scientific, UK
HELIOS	22F6	BioLegend, USA

CD39	A1	BioLegend, USA
GARP	7B11	BioLegend, USA
CTLA-4	BN3	BD Biosciences, USA
TIGIT	1G9	BioLegend, USA
HLA-DR	L243	Thermo Fisher Scientific, UK
CD14	61D3	Thermo Fisher Scientific, UK
CD80	2D10	BioLegend, USA
CD86	2331 (FUN-1)	BD Biosciences, USA
CD163	GHI/61	BD Biosciences, USA
CD206	19.2	Thermo Fisher Scientific, UK
CD40	5C3	BioLegend, USA

Supplementary Figure 1

Representative dot plots (upper panels) showing the gate strategy used to sort M_{exp}, M_{25+} and M_{25-}. Plots (Lower panels) showing the percentages of HLA-A2$^-$ cells before and after sorting

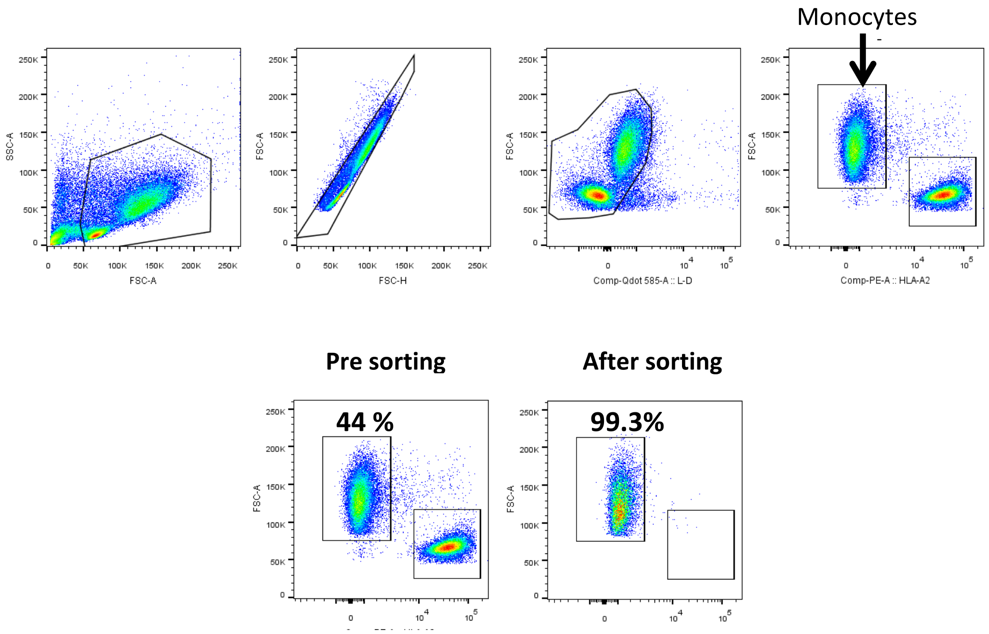

Supplementary Figure 2

Cumulative data showing the percentages of HLA-DR, CD86, CD80, CD14, CD206, CD163 and CD40 in freshly isolated monocytes (n=10)

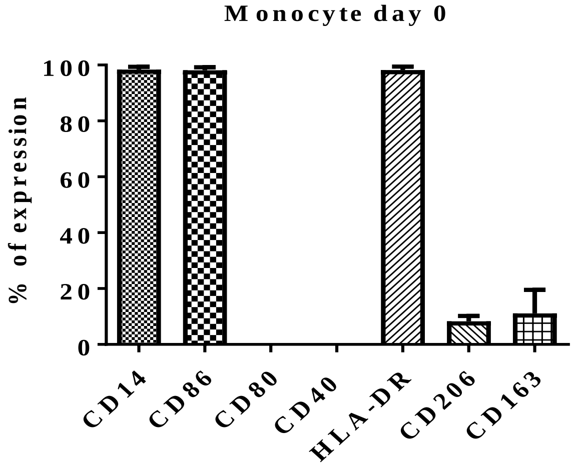

Supplementary Figure 3

Morphology of M_{25+}, M_{25-}, and M_{exp} examinated by light microscopy. One representative image is given.

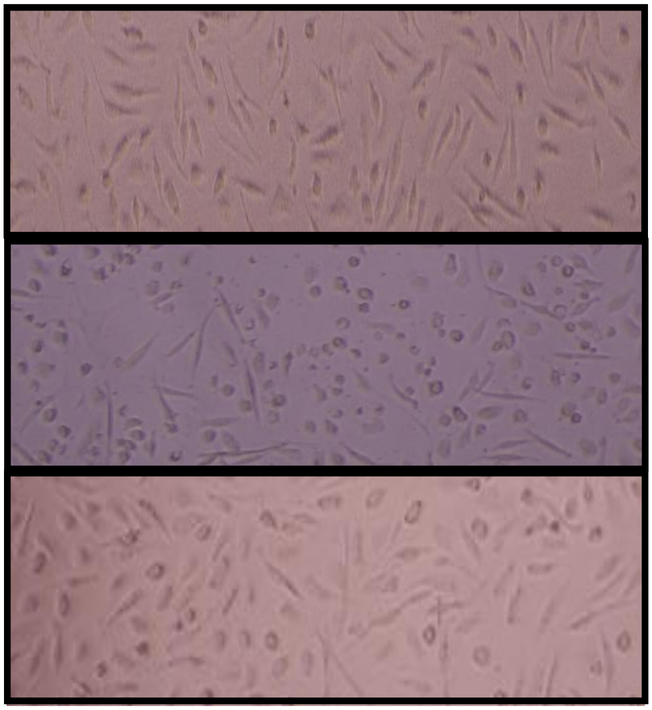

M_{25+}

M_{25-}

M_{exp}

Supplementary Figure 4

Full blots of immunoblots shown in the main figure of the manuscript. The respective figure is indicated.

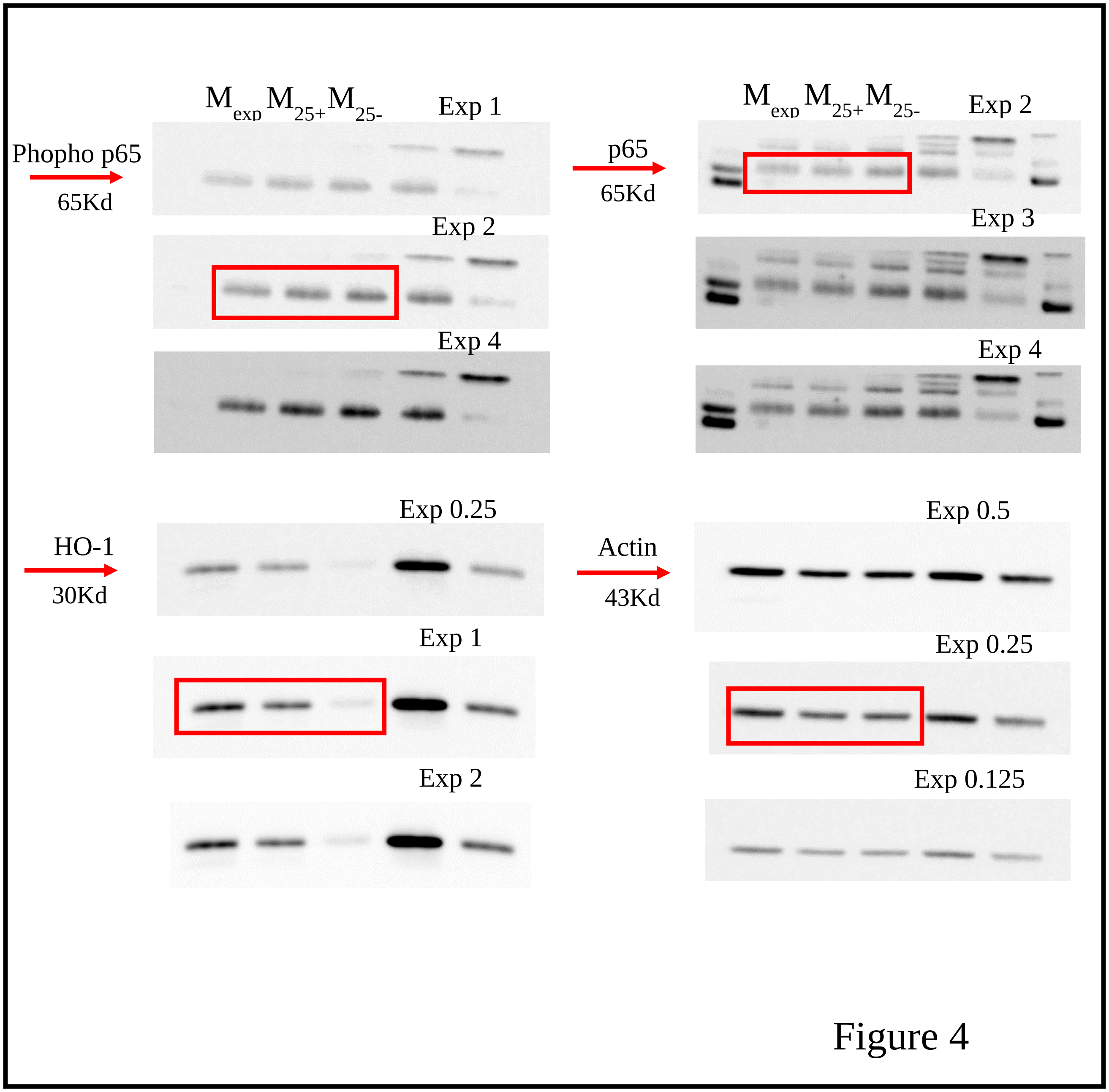

Supplement: Supplementary file 1 [file Data_Sheet_1.PDF]
